# Detecting high‐frequency oscillations in real time during epilepsy surgery with neuromorphic hardware validated to predict postoperative seizure outcome

**DOI:** 10.1002/epi.70400

**Published:** 2026-07-25

**Authors:** Jeroen Teurlings, Filippo Costa, Maeike Zijlmans, Maryse A. van ‘t Klooster, Giacomo Indiveri, Niklaus Krayenbühl, Murezi Capaul, Debora Ledergerber, Georgia Ramantani, Johannes Sarnthein

**Affiliations:** ^1^ Klinik für Neurochirurgie, Universitätsspital Zürich Universität Zürich Zurich Switzerland; ^2^ Institut für Neuroinformatik Universität Zürich and Eidgenössische Technische Hochschule Zürich Zurich Switzerland; ^3^ Department of Neurology and Neurosurgery University Medical Center, Utrecht Brain Center, member of European Reference Network EpiCARE Utrecht the Netherlands; ^4^ Stichting Epilepsie Instellingen Nederland Heemstede the Netherlands; ^5^ Zentrum für Neurowissenschaften Zürich Universität Zürich und ETH Zürich Zurich Switzerland; ^6^ Schweizerisches Epilepsie Zentrum, Klinik Lengg Zurich Switzerland; ^7^ Department of Neuropediatrics and Children's Research Center University Children's Hospital Zurich Zurich Switzerland

**Keywords:** electrocorticography, epilepsy surgery, high‐frequency oscillations, neuromorphic hardware, spiking neural network

## Abstract

**Objective:**

High‐frequency oscillations (HFOs; 250–500 Hz) in electrocorticography (ECoG) are promising biomarkers for delineating the epileptogenic zone during epilepsy surgery. Accurate, intraoperative real‐time detection of HFOs may guide surgical resection to improve seizure outcome.

**Methods:**

We used intraoperative ECoG from 22 patients from Zurich (Nicolet recording device, recording under Sevoflurane) with annotated HFOs to train an innovative automated HFO detector implemented in a spiking neural network (SNN) on neuromorphic hardware. We validated whether the detector could identify clinically relevant HFOs in an independent cohort of 54 patients from Utrecht (Micromed recording device, recording during a pause in propofol administration) by examining whether HFOs remained after resection and whether this predicted seizure outcome. We then tested the detector in real time during surgery, giving direct feedback to the neurosurgeon.

**Results:**

The optimal detection parameters yielded Spearman *R* = .71 between channelwise HFO rates detected by the proposed SNN detection and those previously detected by the Spectrum Detector in the training dataset. We found postresection HFOs in nine patients who had poor surgery outcome and in one patient with good surgery outcome in the validation data, resulting in a diagnostic odds ratio = 17 (95% confidence interval = 2.0–150). During a live surgery, the detector analyzed incoming intraoperative ECoG during acquisition, HFOs were successfully captured, and HFO feedback was available within 1 min after completion of the recording period.

**Significance:**

Our automatic detector was easily transferred between centers and anesthesia protocols. We showed the direct use of intraoperative HFO detection as possible real‐time feedback to the surgeon to tailor resection, yielding high predictive power of seizure outcome.

**Key points**

- We trained the algorithm on intraoperative ECoG of 22 Zurich patients and validated on 54 Utrecht patients.
- Residual HFOs in the validation set predicted poor postoperative seizure outcome (DOR = 17, 95% CI = 2.0–150).
- The external validation across centers and anesthesia protocols demonstrates robustness and generalizability.
- We implemented a spiking neural network on an analog processing chip.
- We detected HFOs in real time within the time limit of surgery and gave feedback to the epileptologist and the surgeon.

## 1 INTRODUCTION

Epilepsy surgery is an established treatment option for selected patients with focal epilepsy.^1^ The neurosurgeon aims to resect the epileptogenic zone, which is defined as the cortex area that can generate epileptic seizures. Total removal of this zone is necessary for seizure freedom. During surgery, intraoperative electrocorticography (ECoG; Figure 1A) may be recorded to optimize the delineation of the epileptogenic zone by analyzing interictal epileptiform patterns, most commonly epileptic spikes.^2^ Spikes are sharply contoured waveforms that occur in the conventional electroencephalographic (EEG) frequency range of <80 Hz. High‐frequency oscillations (HFOs), which are brief bursts of activity in the 80–500‐Hz frequency range, have been proposed as more specific biomarkers of the epileptogenic zone.^3^ Communicating real‐time HFO detection to the neurosurgeon can influence surgical decision‐making, with the aim of enabling more precise tailoring of the resection area.^4^ Traditionally, expert epileptologists visually annotate intraoperative spikes and sometimes HFOs, a process that is time‐consuming and subject to interrater variability.

**FIGURE 1:**
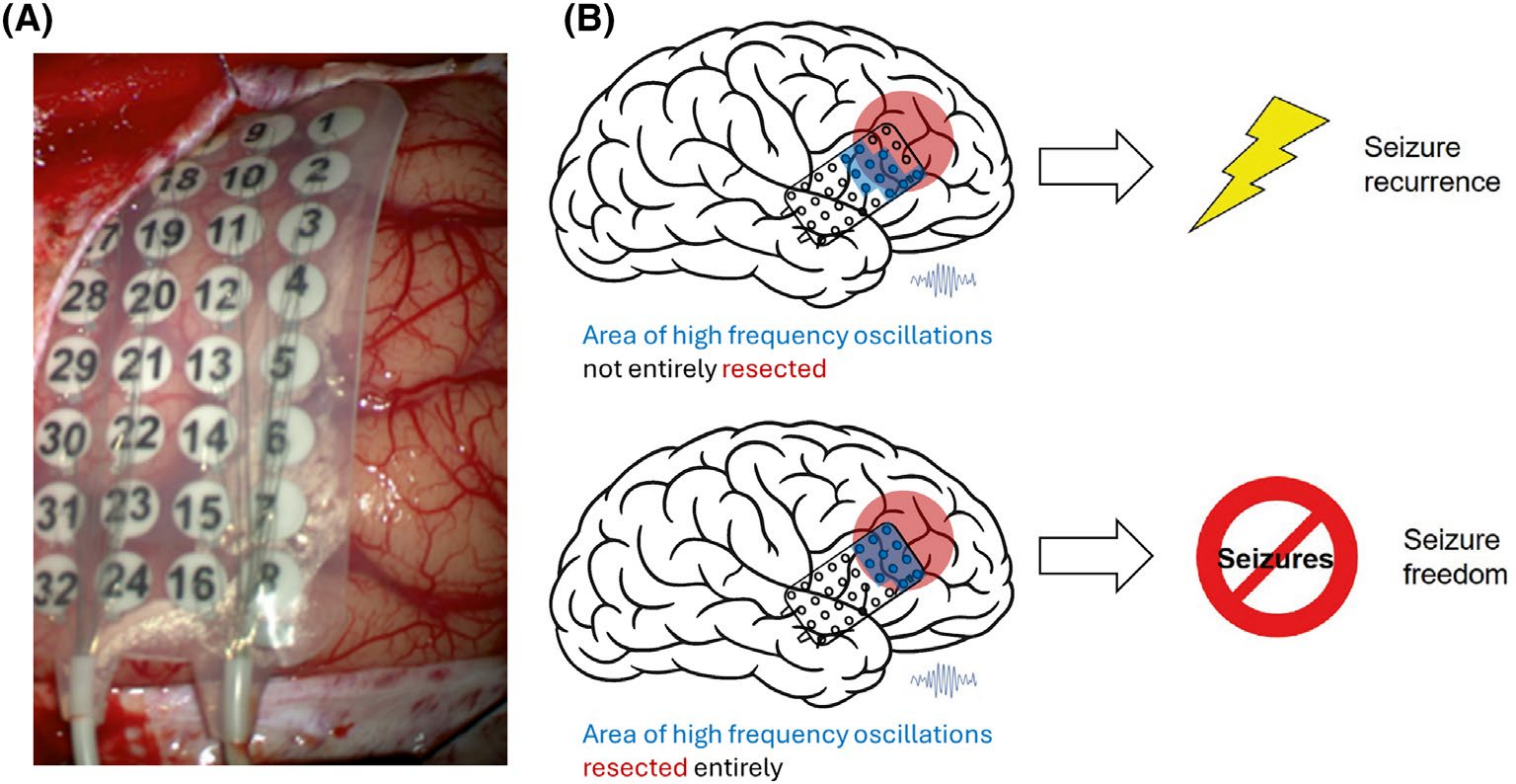
Intraoperative recordings. (A) During epilepsy surgery, the neurosurgeon places an electrode on the cortex to record electrocorticography. (B) We hypothesize that high‐frequency oscillations (HFOs) indicate the epileptogenic zone. Total removal of the epileptogenic zone is necessary for seizure freedom. Total resection of the HFO area results in postoperative seizure freedom (good seizure outcome). Conversely, if at least one channel with HFOs is not resected, the patient will suffer from postoperative seizure recurrence (poor seizure outcome).

Several automated HFO detectors have been developed,^5^, ^6^, ^7^, ^8^ and some are capable of real‐time HFO detection.^9^, ^10^ Automated detectors not only reduce the time and effort required from expert reviewers but can also be trained and validated against postoperative seizure outcomes using large, prerecorded datasets (Figure 1B).^11^ There is an ongoing debate on which specific patterns in the HFO frequency range are relevant for outcome prediction.

We present an algorithm for HFO detection based on a spiking neural network (SNN) implemented on neuromorphic hardware.^12^, ^13^ Neuromorphic hardware offers advantages such as low power consumption, robust signal compression, and real‐time processing. Although power consumption is not a limiting factor during surgery, rapid HFO detection within the surgical timeframe is important for providing timely feedback to the neurosurgeon.

Building on our earlier work,^9^, ^14^ which focused on technical feasibility, we here demonstrate clinical translation through outcome prediction, external validation, and intraoperative proof‐of‐concept. We applied a new algorithm that allowed straightforward parameter optimization.^15^ We trained this algorithm on an annotated HFO dataset,^7^, ^16^ validated its performance by predicting postoperative seizure outcomes in an external cohort,^5^, ^17^ and then detected HFOs in real time during epilepsy surgery.

## 2 MATERIALS AND METHODS

### 2.1 Patients and datasets

We used data from three separate sources of intraoperative subdural ECoG recordings from patients with drug‐resistant epilepsy. In all datasets, intraoperative ECoG was recorded with subdural disk electrodes. We divide the data into a training set, a validation set, and a test case.

#### 2.1.1 Training dataset

The training dataset (Table S1) comprises 22 patients who underwent epilepsy surgery at Universitätsspital Zurich (USZ) between 2015 and 2018.^7^, ^16^ For each patient, pre‐ and postresection recordings were made under inhalational anesthesia (Sevoflurane) with electrode contact spacing of 5 mm or 10 mm, resulting in 44 recordings collected with a Nicolet device (sampling rate = 2 kHz, 1–800‐Hz passband). This dataset includes HFO annotations that had been created automatically with an algorithm that we refer to as Spectrum Detector.^7^

#### 2.1.2 External validation dataset

The validation dataset (Table S2) included 54 patients who underwent epilepsy surgery at Universitair Medisch Centrum Utrecht (UMCU) between 2008 and 2012,^17^ with ECoG recorded during a pause in propofol administration from electrode grids with contact spacing of 10 mm.^18^ Data were collected using a 64‐channel EEG system (Micromed) at a 2048‐Hz sampling rate with a 538‐Hz antialiasing filter. We used the 54 postresection recordings from this dataset to validate HFO detection against postoperative seizure outcome.

#### 2.1.3 Test case

We tested the validated real‐time HFO detection during epilepsy surgery in one patient at USZ. The data were acquired during surgery and analyzed during acquisition. Recordings were made under inhalational anesthesia (Sevoflurane, minimum alveolar concentration < .5) with a Micromed EEG system at 2048‐Hz sampling frequency and a 538‐Hz antialiasing filter.

### 2.2 Preprocessing and asynchronous delta modulator encoding

All ECoG data were rereferenced to a bipolar montage before filtering and HFO analysis. We filtered the raw ECoG signals in the fast ripple band (250–500 Hz) and then converted the filtered signals into discrete digital pulses using asynchronous delta modulator (ADM) encoding implemented in Python (Figure 2). This process transformed the continuous time series into discrete asynchronous UP and down (DN) pulses (Figure 3A). For each channel, the ADM threshold (the minimal amplitude change in the signal to produce an UP or DN pulse) was determined adaptively; the first second of the signal was divided into 10 segments, the amplitude (maximum minus minimum value) of each segment was calculated, and the 25th percentile of these amplitudes was selected as the ADM threshold. This approach ensured that the encoding accounted for each channel's individual noise level.

**FIGURE 2:**
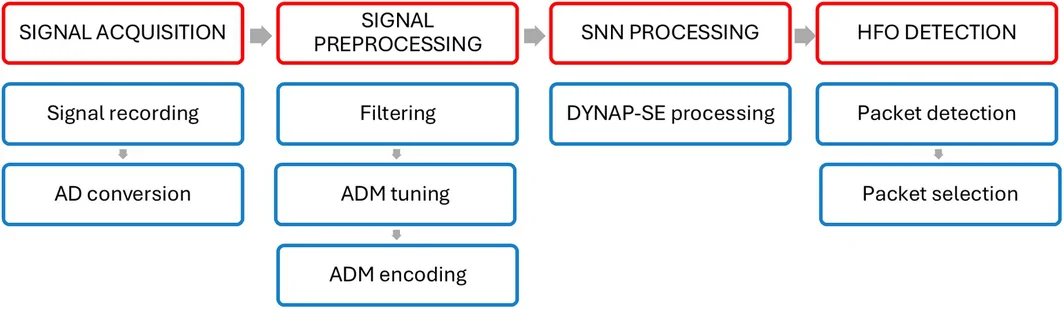
Data analysis workflow for neuromorphic high‐frequency oscillation (HFO) detection. The detection of HFOs consists of four steps. (1) Signal acquisition: We recorded electrocorticography (ECoG) during surgery and saved the digitized signal traces. (2) Signal preprocessing in Python: We filtered the ECoG (250–500 Hz). We tuned the parameters of the asynchronous delta modulator (ADM) during the first second of recording and then encoded the ECoG traces into discrete UP and down (DN) pulses. (3) Neuromorphic processing: UP/DN pulses were streamed into the spiking neural network (SNN) implemented in the Dynamic Asynchronous Neuromorphic Processor (DYNAP‐SE) chip. (4) HFO detection: We defined packets in the stream of SNN output pulses. If the parameters of a packet were within the optimal limits, we marked it as an HFO. AD, analog–digital.

**FIGURE 3:**
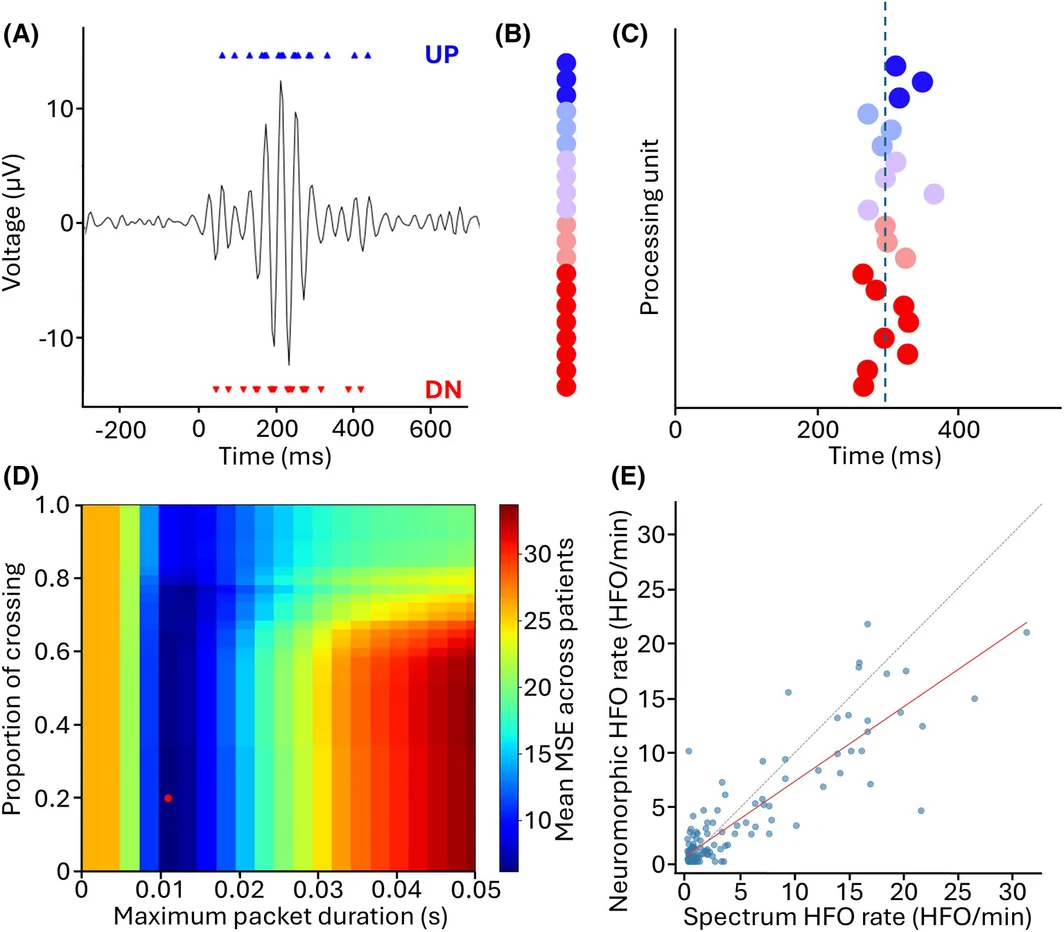
Neuromorphic packet detection in the training data. (A) The filtered electrocorticographic (ECoG) signal was converted through asynchronous delta modulator encoding into UP and down (DN) pulses (blue and red triangles). Each ECoG channel was assigned to a group of spiking neural network (SNN) processing units. Connections between the UP stream, DN stream, and individual processing units were assigned at random. (B) The group of SNN processing units within the chip. They vary in their preference for UP input (shades of blue) or DN input (shades of red). This heterogeneous mix of processing units ensures a heterogeneous population response. (C) Packets are bursts of population activity where each processing unit is allowed to fire at most one pulse (colored dot). In a packet, the pulse times of the processing units are referenced to the median firing time (dashed line). (D) After varying two parameters in a grid search for the optimal combination, we achieved minimal mean standard error (MSE) between the high‐frequency oscillation (HFO) rate from the SNN and the Spectrum Detector in the training set^5^ (red dot, MSE = 6.2 HFO/min; optimal packet parameters: proportion of crossings = .2, maximum packet duration = .01 s). (E) Comparison of channelwise HFO rates between the Spectrum Detector and the Neuromorphic Detector. Each channel is a dot, and we plotted the HFO rate for both detectors. The linear fit (red line) has a slope of .68 (95% confidence interval = .62–.75, Spearman *R* = .71), indicating that the Neuromorphic Detector marks fewer HFOs than the Spectrum Detector.

### 2.3 Hardware processing

The stream of UP and DN pulses was sent to the Dynamic Asynchronous Neuromorphic Processor (DYNAP‐SE), which contains analog processing units in four cores and uses pulse‐based processing. Each ECoG channel was assigned to an SNN with a single layer of processing units on the chip. The number of processing units per channel was determined by dividing the maximum number of units per core (256) by the number of channels. This ensured that the largest number of units was used per channel.

Connections between the UP/DN streams and the individual processing units were made at random, keeping the maximum total number of connections fixed. This resulted in processing units that varied in their response to UP input or DN input (Figure 3B). Due to the inherent properties of the hardware, these units have heterogeneous response properties. When the ECoG signal is injected into the SNN, the group of *n* processing units produces population bursts with at most one pulse per unit. We defined these bursts as “packets,” short bursts of output pulses from the SNN processing units that formed the basic event candidates from which HFOs were selected.^15^ For each packet, we encoded the pulse times of all processing units relative to the population median, resulting in an *n*‐dimensional vector representation (Figure 3C).

The hardware parameters were calibrated once so that the SNN response matched the typical time scale of HFOs (10–20 ms^9^). Synaptic weights were set to produce SNN output in response to one reference HFO snippet. This calibration ensured that HFO input produced packets of output pulses.

### 2.4 Parameter optimization

To select HFOs from the packet pool, we defined detection rules. We used the training dataset to optimize the hardware and packet parameters so that the detected HFO rates reproduced the HFO rates obtained with the validated Spectrum Detector.^7^ The packet selection was optimized using two parameters: (1) the maximum duration of the packet and (2) the minimum proportion of UP/DN crossings within a packet (Figure 3D).^15^ Low maximum duration of the packet excludes most packets captured by the chip. High maximum duration allows most packets to be accepted as HFOs. The proportion of UP/DN crossings quantifies how often UP‐ and DN‐related output pulses alternate within a packet. A value of 1 indicates complete alternation between UP‐ and DN‐related output pulses, whereas a value of 0 indicates that all pulses in a packet are related to only one input direction.

For each recording in the training set, we performed a grid search varying the proportion of crossings between 0 and 1 in steps of .025 and the maximum duration of the packets between 0 s and .05 s in steps of .0025 s, calculating the mean standard error (MSE) between the HFO rates of the Neuromorphic and Spectrum detectors for each grid point. Recordings where the Spectrum Detector detected zero HFOs were excluded from training. The MSE per combination of parameters was averaged across all patients. Packets with parameters within these limits were labeled as HFOs. As a last step in the detection pipeline, we removed detected HFOs that occurred within 1 s of each other to remove double detections.

### 2.5 HFO rate and HFO area

We analyzed the validation dataset^17^ with the optimal parameter values. The detector does not explicitly distinguish oscillatory HFOs from sharp transients that mimic oscillations after filtering. The events marked by the detector were validated as clinically relevant HFOs by their association with postoperative seizure outcome. We converted the HFOs found using this packet‐based method into rates by dividing the number of detected HFOs by the total recording time in minutes and the number of channels with detected HFOs (HFO rate: HFO/min/channel with HFO).

$$\mathrm{HFO}\;\text{rate}\;\left(1/\min\right)=\frac{\text{detected HFOs}}{\text{channels with HFOs}\times \text{total time}\;\left(\min\right)}$$

Detection results were visually checked for obvious false detections due to, for example, whole‐grid high‐amplitude noise, or electrode contacts detached from the brain. Visual artifact assessment was used to exclude clearly unstable recording periods. We did not reject individual detector‐marked HFOs during stable recordings. The group of channels with an HFO rate of >1 HFO per minute defined the HFO area, as clinical outcome prediction is most accurate when using a threshold of >1 HFO per channel per minute.^5^ This threshold is practical for surgeons, who are more likely to adjust the resection strategy if more than 1 HFO per minute is detected than with only 1 HFO per minute, as sometimes the artifact‐free recording period is only 1 or a few minutes long. We used Spearman correlation and a linear fit to compare HFO rates between detectors.

### 2.6 Performance metrics

We validated HFO detection by analyzing the association between residual HFOs and postoperative seizure outcomes in the validation set.^17^ Our hypothesis was that the HFOs relate to epileptogenic tissue; complete removal of the cortex generating HFOs results in seizure freedom, whereas incomplete removal with residual HFOs leads to recurrent seizures (Figure 1B). In the validation set, postoperative outcomes were classified using the Engel classification and dichotomized into good outcome (complete seizure freedom, Engel IA) and poor outcome (recurrent seizures, Engel IB–IV).^17^ For the confusion matrix,^3^ true positives (TP) were patients with residual HFOs after resection and poor outcome; true negatives (TN) were patients without residual HFOs and good outcome; false positives (FP) were patients with residual HFOs but good outcome; and false negatives (FN) were patients without residual HFOs but poor outcome. We evaluated diagnostic performance by calculating sensitivity, specificity, positive predictive value (PPV = TP / [TP + FP]), negative predictive value (NPV = TN / [TN + FN]), likelihood ratios (LR+ = sensitivity / [1 – specificity] and LR– = [1 – sensitivity] / specificity), and diagnostic odds ratio (DOR = LR+ / LR–). Additional metrics included true positive rate (TPR = TP / [TP + FN]), false positive rate (FPR = FP / [FP + TN]), accuracy ([TP + TN] / *n*), and area under the curve (AUC = [1 + TPR + FPR] / 2). Sensitivity was defined as TPR and specificity as 1 – FPR. For each metric, 95% confidence intervals (CIs) were constructed using the Wilson method with the binomial distribution. The detector was evaluated based on its predictive power for seizure outcome, which is the ultimate goal in optimizing the definition of an HFO in the signal.

### 2.7 Real‐time HFO detection during epilepsy surgery

To test the proposed method of HFO detection in real time, ECoG was recorded in the surgical theater with a 1 × 4 contact point electrode subdural strip (AdTech Medical) that contains platinum electrode contact points encased in a silicone strip. The electrode points had a contact exposure diameter of 2.3 mm and an interelectrode distance of 10 mm. A needle electrode placed in the dura was used as a common reference point. We filtered the raw ECoG signal (finite impulse response [FIR], 250–500 Hz) and streamed it to an external laptop for data analysis through the BCI2000 framework with the MicromedADC module.^19^

We applied ADM to encode the filtered signal into UP/DN pulses (Figure 3A). These pulses were input to the SNN in the DYNAP‐SE neuromorphic chip. The SNN generated output pulses, which were grouped into packets (Figure 3B,C). Packets with parameters within the optimized limits were marked as HFOs.

After surgery, we visually inspected the ECoG data (FIR filtered in Python, 250–500 Hz) to confirm the presence of HFOs in the data (Figure 5).

## 3 RESULTS

We will first describe the optimization of the detector parameters in the training set, then the validation of the detector in the validation set and finally its performance in real‐time detection.

### 3.1 Parameter optimization in training data

We optimized the parameters to minimize the MSE between the HFO rates detected by the Spectrum Detector (training set) and the Neuromorphic Detector (Figure 3D). This resulted in an average heatmap showing the optimal combination of values across all patients in the training set. The lowest MSE (6.2 HFO/min) was achieved with a proportion of crossings of .2 and a maximum packet duration of .01 s. To illustrate the agreement between detectors, we plotted the HFO rate for each channel as marked by both the Neuromorphic Detector and the Spectrum Detector (Figure 3E), finding a high correlation (Spearman *R* = .71, *p* = 1e‐21). The slope of the linear fit (.68, 95% CI = .62–.75) indicates that the Neuromorphic Detector marks fewer HFOs than the Spectrum Detector.

### 3.2 Performance of the HFO detector in validation data

The general settings of the chip and the packet parameters were fixed and used to identify HFOs in the validation set (Figure 4A). When investigating the HFO rates marked by the Neuromorphic Detector and Visual Annotation, we found a high correlation (Spearman *R* = .79, *p* = 6e‐5). The slope of the linear fit (.30, 95% CI = .18–.41) indicates that the Neuromorphic Detector marks much fewer HFOs than Visual Annotation.

**FIGURE 4:**
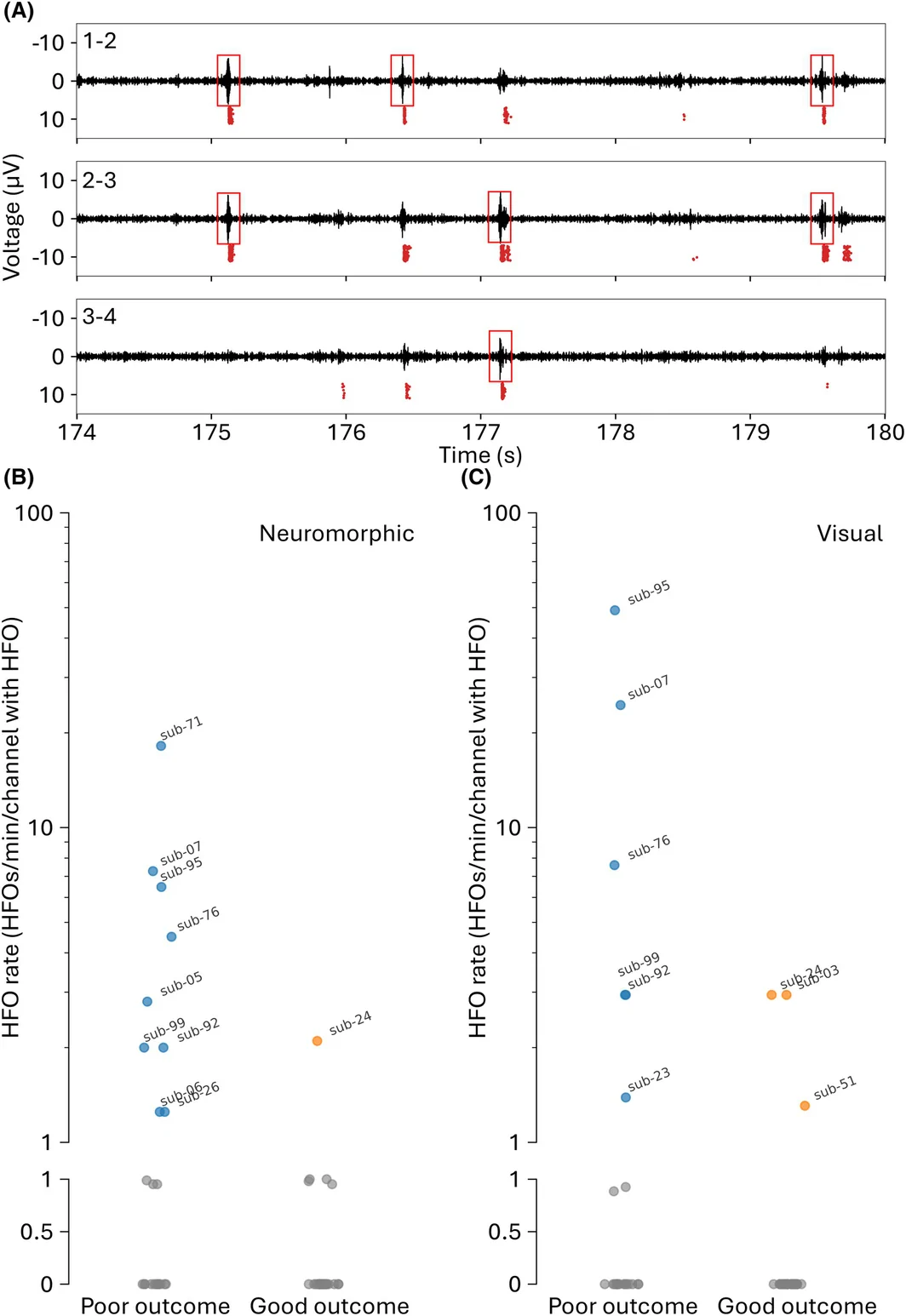
High‐frequency oscillation (HFO) rate and postoperative seizure outcome in the external validation dataset with the Neuromorphic Detector. (A) Events in three closely located electrocorticographic channels (black, 6‐s duration) elicit spiking neural network output pulses (red dots). Only the packets of pulses with parameters inside the optimized limits were marked as HFOs (red boxes) in the validation dataset.^17^ (B) Neuromorphic HFO detection. Nine patients with residual HFOs suffered from recurrent seizures (poor surgery outcome, blue dots), and one patient with residual HFOs achieved postoperative seizure freedom (good surgery outcome, orange dot). Gray dots depict rates below the threshold of 1 HFO per minute. (C) Visual HFO markings. Six patients with HFO had poor outcome, and three patients with HFO had good outcome. Good outcome = Engel IA, poor outcome = Engel IB–IV. “sub‐” precedes the patient number.

We evaluated the Neuromorphic Detector by assessing its diagnostic performance in predicting seizure outcome in the validation set (*n* = 54; 24 patients with poor outcome and 30 with good outcome).^17^ We identified residual HFOs in nine patients with recurrent seizures and one patient with postoperative seizure freedom (Figure 4B, Table S2), which yields nine TP, 29 TN, 1 FP, and 15 FN detections (Table 1). In the original analysis of the validation set using visual HFO annotation, residual HFOs were found in six patients with poor outcome and three patients with good outcome (Figure 4C). The derived diagnostic metrics are presented in Table 2. DOR = 17 (95% CI = 2.0–150) reflects the detector's diagnostic performance, versus DOR = 5.4 (95% CI = 1.3–23) for visual analysis.

**TABLE 1:** Confusion matrix for neuromorphic HFO detection in the external validation dataset.

	Residual HFOs	No residual HFOs
Postoperative seizures (poor outcome), *n* = 24	TP = 9	FN = 15
Seizure‐free (good outcome), *n* = 30	FP = 1	TN = 29

Abbreviations: FN, false negative, no residual HFOs but patient is not seizure‐free; FP, false positive, patient with residual HFOs but seizure‐free; HFO, high‐frequency oscillation; TN, true negative, patient without residual HFOs and postoperatively seizure‐free; TP, true positive, patient with residual HFOs and not seizure‐free.

**TABLE 2:** Association between residual HFOs and postoperative seizure recurrence.

	Neuromorphic Detector [95% CI]	Spectrum Detector [95% CI]^5^	Visual Annotation [95% CI]^17^
TPR, sensitivity	38% [21%–57%]	25% [12%–45%]	38% [21%–57%]
1 − FPR, specificity	97% [83%–99%]	100% [89%–100%]	90% [74%–97%]
PPV	90% [67%–98%]	100% [61%–100%]	75% [47%–91%]
NPV	66% [51%–78%]	62% [48%–75%]	64% [49%–77%]
Accuracy	70% [57%–81%]	66% [53%–78%]	67% [53%–78%]
AUC	70%	62%	64%
LR+	11 [1.3–95]	NaN	3.7 [.9–15]
LR–	.6 [.3–1.5]	.7 [.3–1.7]	.7 [.3–1.6]
DOR	17 [2.0–150]	NaN	5.4 [1.3–23]

*Note*: Metrics are derived from the confusion matrix for the validation set with *n* = 54 patients.^17^ PPV = TP / (TP + FP), NPV = TN / (TN + FN), TPR = TP / (TP + FN), FPR = FP / (FP + TN), accuracy = (TP + TN) / *n*, AUC = (1 + TPR + FPR) / 2, sensitivity = TPR, specificity = 1 − FPR, LR+ = sensitivity / (1 − specificity), LR– = (1 − sensitivity) / specificity, DOR = LR+ / LR–. For better comparison, some entries in the table were calculated from the original values in the cited publications.^5^, ^17^

Abbreviations: AUC, area under the curve; CI, confidence interval; DOR, diagnostic odds ratio; FN, false negative; FP, false positive; FPR, false positive rate; LR–, negative likelihood ratio; LR+, positive likelihood ratio; NaN, not a number; NPV, negative predictive value; PPV, positive predictive value; TN, true negative; TP, true positive; TPR, true positive rate.

### 3.3 Real‐time HFO detection during epilepsy surgery

We recorded from a 26‐year‐old female patient with a structural lesion and focal seizures due to a focal cortical dysplasia type IIa in the right postcentral cortex who underwent ECoG‐guided epilepsy surgery at USZ.

The raw ECoG recordings from the clinical EEG system were streamed to a secondary laptop, where an ADM module encoded the signals into UP/DN pulses. The pulses were then sent to the neuromorphic chip (Figure 2). The resulting pulses from the chip were further analyzed in Python to detect packets and identify HFOs. The analysis pipeline from ECoG recording to HFO detection was completed during the course of the surgery with successful HFO detection as shown in Figure 5. In the intraoperative test case, recording periods lasted 2–6 min. HFOs were marked within .1 s of their occurrence, and final HFO rates were available approximately 1 min after the end of the recording period.

**FIGURE 5:**
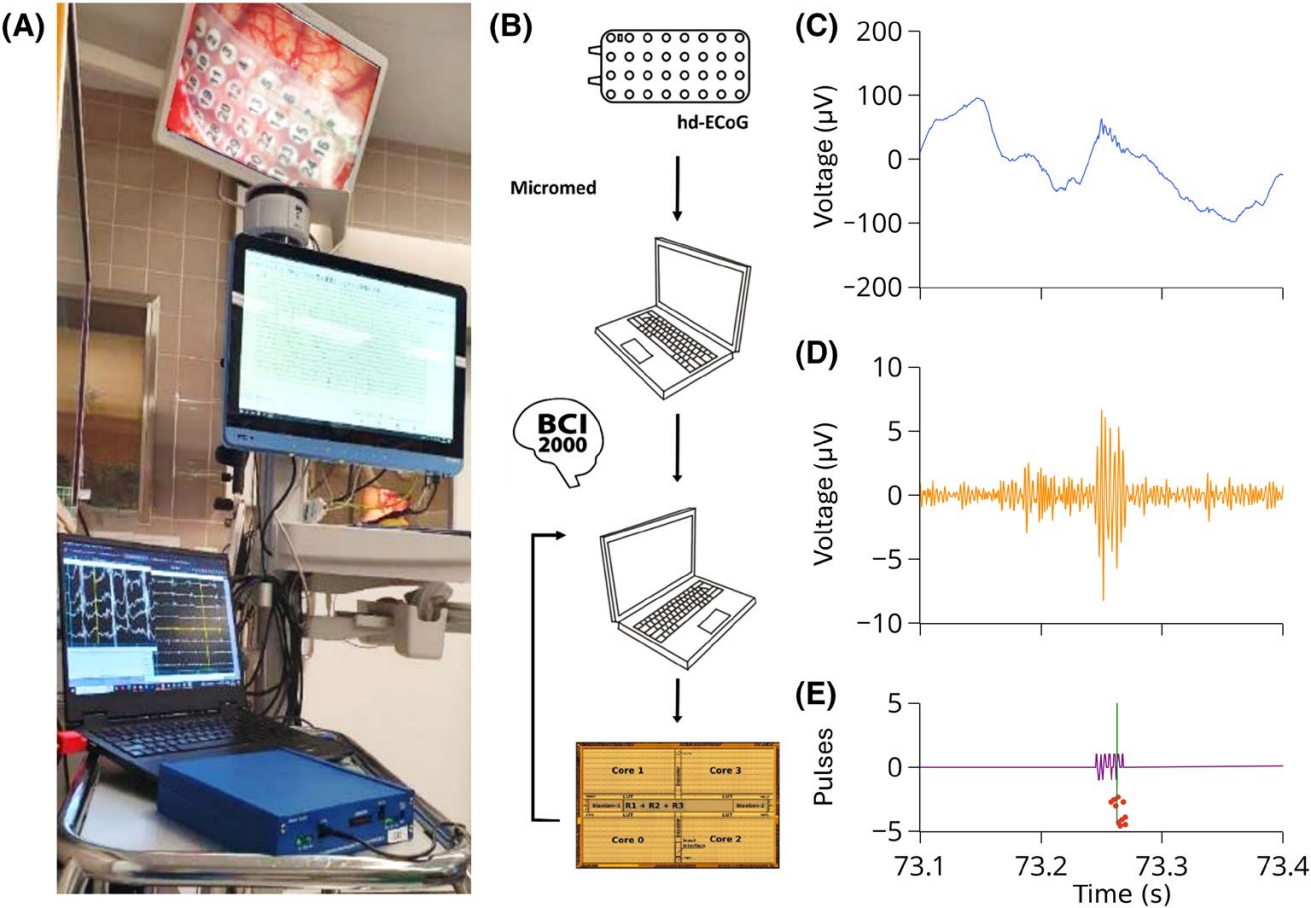
High‐frequency oscillations (HFOs) detected in real time during epilepsy surgery (test case). (A) Setup of the detection method during surgery. Electrocorticogram (ECoG) is intraoperatively recorded with a Micromed device, streamed to an external laptop, filtered, and sent to the neuromorphic chip. (B) Schematic overview of the detection setup. (C) Broadband ECoG signal as measured by the Micromed device. (D) Filtered ECoG signal between 250–500 Hz. An HFO is visible. (E) Asynchronous delta modulator encoding of the filtered signal results in up/down pulses (violet trace) that enter the spiking neural network (SNN). The SNN emits pulses (red dots), which indicate HFO detection (green line). hd, high density.

## 4 DISCUSSION

We present a neuromorphic, packet‐based HFO detector that transforms intraoperative ECoG signals into ADM‐encoded UP/DN pulses, thereby compressing the data and leveraging heterogeneous pulse responses on the DYNAP‐SE neuromorphic hardware. HFOs were detected by clustering SNN pulses into packets, with parameters trained on data from 22 patients in the training set. We then validated the performance of the trained network on 54 patients in the validation set by comparing detected residual HFOs with postoperative seizure outcomes. Residual HFOs after resection were associated with poor seizure outcome both in Neuromorphic Detection and Visual Annotation (Table 2). The successful application of the detection method during an epilepsy surgery demonstrated the feasibility of real‐time HFO detection.

### 4.1 Variations in ECoG recording settings

We compared ECoG recordings obtained under different settings routinely used in the two epilepsy surgery centers in Zurich and Utrecht, including differences in clinical amplifier systems, inherent filtering, sampling frequency, and electrode contact distance. Additionally, the anesthesia regimen differed; the training set was recorded under inhalational anesthesia (Sevoflurane), whereas the validation set was recorded after pausing propofol.^18^, ^20^ Despite these variations, the trained parameter values yielded good seizure outcome prediction in the validation set, indicating that our Neuromorphic Detector with its signal preprocessing steps can perform well in different surgical settings. This external validation across centers and anesthesia protocols demonstrates robustness and generalizability and supports its potential use in various epilepsy surgery centers.

### 4.2 Real‐time HFO detection during surgery

We implemented the HFO detection pipeline during epilepsy surgery, demonstrating that detection can be completed within the timeframe of the procedure. Data streaming and analysis with the neuromorphic chip occur in real time, allowing spiking outputs to be generated with minimal delay. These outputs are rapidly grouped into packets and filtered to detect HFOs, supporting the feasibility of routine intraoperative monitoring of HFOs in ECoG. As a limitation of our study, we will only be able to report seizure outcome of the surgery with real‐time HFO detection (Figure 5) after 24‐month follow‐up. Clinical validation of our algorithm is restricted to prerecorded data (Figure 4), whereas the live test case functions solely as a proof‐of‐concept for intraoperative real‐time application of the proposed method.

### 4.3 Advantages of neuromorphic hardware

The neuromorphic implementation enables real‐time, event‐based processing of intraoperative ECoG with a compact detection pipeline. The packet‐based detection algorithm can be optimized with only two parameters, facilitating adaptation to other chips and surgical settings.

The neuromorphic method also reduces data size, as the DYNAP‐SE chip transmits only neuron firing times rather than the entire signal. Employing shallow networks enables pattern detection based on sparse firing patterns from a single layer, which reduces power consumption compared to software‐based analyses. Although power requirements are not a concern in the operating theater, low‐power methods could be advantageous in power‐restricted scenarios such as wearable or implanted devices.

Because HFO detection relies on the heterogeneous response of the hardware to input, the approach can be easily adapted to other neuromorphic hardware platforms.^12^, ^13^ The pattern detection algorithm^15^ is also applicable to other use cases by tuning circuit parameters and detection rules for different epileptiform patterns. Rather than requiring manual tuning of hardware biases, the SNN can be optimized with a single tuning snippet for each pattern of interest, offering an advantage over standard machine learning approaches that require large training datasets. This method produces an efficient, sparse temporal encoding that enables the design of simple detection rules by explicitly using device mismatch.

We explicitly distinguish this study from our earlier work,^9^, ^14^ which focused on technical feasibility. We here demonstrate clinical translation through outcome prediction, external validation, and intraoperative proof‐of‐concept. By reducing optimization to only two parameters, the packet‐based algorithm is fundamentally distinct from the prior architecture, offering a simplified and practical solution for clinical adoption.

### 4.4 Clinical implications

Our work represents a step forward in bringing automatic HFO detection into the operating theater. HFOs occur infrequently, with maximum rates of only a few per minute, as observed in both visual and automatic detections (Figure 4). They are also brief (tens of milliseconds) and low amplitude (microvolts), making accurate detection demanding in terms of time and resources.^3^, ^11^ Reliable detection is promoted by optimized recording paradigms, including high electrode density ECoG grids,^7^ and effective communication among the surgical team. Here, we present a new, robust method for intraoperative HFO detection.

Direct HFO detection during surgery may be able to aid epilepsy surgery, especially for extratemporal lobe epilepsy.^3^ The first prospective attempt, the HFOtrial,^4^ encountered practical limitations like surgical artifacts caused by surgical equipment. This trial tested a narrow question (seizure freedom with HFOs versus spike tailoring), and the methodology (power and randomization) appeared not equipped for the heterogenicity in patient factors like size of resection and underlying pathology. A correction for these factors led to the conclusion that careful interpretation of HFOs is warranted for temporal lobe epilepsy, enforced by simultaneous findings that HFOs in the hippocampus are often physiological and temporal lobe HFOs are merely an indirect outcome predictor in hippocampus sparing temporal lobe resections.^21^ The corrected HFOtrial results showed that HFOs were noninferior to spikes in extratemporal epilepsy surgery, and similar good predictive values of intraoperatively measured HFOs were found in extratemporal lobe epilepsy studies.^3^

The Neuromorphic Detector shows similar diagnostic performance metrics to visual marking (Table 2). Although the DOR = 17 (95% CI = 2.0–150) has a wide CI, even at the lower bound of the CI, a positive test result (i.e., detection of HFOs) doubles the probability of postoperative seizures. The wide CI results from the relatively high number of FN. It is important to note that these FN do not solely reflect the detector's performance. They also depend on the extent of the recording surface during surgery. If the ECoG grid does not cover all cortical regions where HFOs are present, the detector cannot identify them, leading to a higher number of FN.

Compared to Visual Annotation, the Neuromorphic detector marked only one third of the patterns in the validation dataset as clinically relevant HFOs, whereas the diagnostic performance metrics were equally high. Although this may suggest that the Neuromorphic Detector is more specific in marking outcome‐relevant HFOs, a systematic event‐level comparison was beyond the scope of this study, which focuses on the association of HFO rates with postoperative seizure outcome.

We excluded recording periods that, upon visual inspection, were attributable to obvious artifacts such as electrode grid detachment or high‐amplitude noise from intraoperative activity. This process reflects the intended use of the Neuromorphic Detector as an assistant to the monitoring team by providing additional standardized information during surgery, rather than as a stand‐alone marker or a replacement for spike‐based expert intraoperative localization of the epileptogenic zone.^11^, ^22^ By incorporating an expert‐in‐the‐loop workflow, where epileptologists review and select HFOs, the neuromorphic platform can serve as a semiautonomous assistant, reducing the human workload in epileptiform pattern annotation while still allowing for expert oversight.

### 4.5 Future perspectives

We present a first example of real‐time HFO analysis in the operating theater to provide real‐time assistance to the surgeon based on detected HFOs. A key advantage of the neuromorphic approach to HFO detection is its low power requirement.^12^, ^13^ Although power consumption is not a concern in the operating theater, it becomes critical for wearable or implanted devices, where low‐power analysis extends battery life and reduces the need for frequent charging. Our automated approach could enable real‐time clinically relevant HFO detection suitable for intraoperative monitoring for epilepsy surgery. An easy availability of intraoperative HFO detection might spread its use among epilepsy centers.

## 5 CONCLUSIONS

The external validation against postsurgical seizure outcome across centers and anesthesia protocols demonstrates robustness and generalizability of the automated algorithm. The proposed intraoperative HFO detection was readily optimized with only two parameters. It can, in principle, be used as actionable feedback to the neurosurgeon to tailor resection. A first case of real‐time HFO detection opens the path to further research on real‐time intraoperative epileptogenic zone delineation using HFOs.

## AUTHOR CONTRIBUTIONS

**Jeroen Teurlings:** Writing—original draft; software; visualization. **Filippo Costa:** Methodology; data curation; software. **Maeike Zijlmans:** Data curation; writing—review & editing. **Maryse A. van ‘t Klooster:** Data curation; writing—review & editing. **Giacomo Indiveri:** Methodology. **Niklaus Krayenbühl:** Data curation. **Murezi Capaul:** Software. **Debora Ledergerber:** Methodology; **Georgia Ramantani:** Data curation; writing—review & editing. **Johannes Sarnthein:** Writing—original draft; conceptualization; funding acquisition; supervision. All authors reviewed and approved the final version of the manuscript.

## CONFLICT OF INTEREST STATEMENT

None of the authors has any conflict of interest to disclose.

## ETHICS STATEMENT

The study was approved upfront by the institutional ethics review boards of the two centers according to the Declaration of Helsinki (Kantonale Ethikkommission Zürich, 2019‐01977, RESPect database, UMCU MREC 18‐109C). The collection of patients' written informed consent was waived in Zurich, and Utrecht's institutional ethics committee waived the need for informed consent for all retrospectively collected data before 2018 (as of 2018 informed consent is required and obtained) and approved the use of pseudonymized data in the RESPect database for the purpose of retrospective research. We confirm that we have read the Journal's position on issues involved in ethical publication and affirm that this report is consistent with those guidelines.

## CODE AVAILABILITY STATEMENT

Additional data and code are indexed at www.hfozuri.ch.

## Supporting information


**Supplementary Table 1** Seizure outcome of the patients in the training set, their outcome classification, and their postresection HFO rate. Good = ILAE 1, poor = ILAE > 1. DNET, dysembryoplastic neuroepithelial tumors; FCD, focal cortical dysplasia; HFO, high‐frequency oscillation; ILAE, International League Against Epilepsy; ODG, oligodendroglioma.
**Supplementary Table 2** Seizure outcome of the patients in the validation set and their outcome classification. The outcome prediction yields the elements of the confusion matrix for the validation set (Table 1).^16^ DNET, dysembryoplastic neuroepithelial tumor; f, female; FCD, focal cortical dysplasia; FN, false negative; FP, false positive; Good, outcome Engel IA; m, male; MCD, mild cortical dysplasia; MTS, mesial temporal sclerosis; Poor, outcome Engel IB–IV; TN, true negative; TP, true positive; TSC, tuberous sclerosis.

## Data Availability

All data needed to evaluate the conclusions in the paper are present in the paper. The training data set is freely available at https://doi.org/10.18112/openneuro.ds004944.v1.0.1.^7^, ^16^
