# Supplementary material for: Detecting high‐frequency oscillations in real time during epilepsy surgery with neuromorphic hardware validated to predict postoperative seizure outcome
Source: Epilepsia. 2026 Jul 25;67(10):5215–26. doi: 10.1002/epi.70400 (PMC13675772; doi:10.1002/epi.70400)
Supplement: Supplementary file 1 [file EPI-67-5215-s001.docx]

Supplementary Table 1. Seizure outcome of the patients in the training set and their outcome classification. Patients in the training set with seizure outcome and post-resection HFO rate. Good = ILAE 1, Poor = ILAE > 1. FCD = focal cortical dysplasia; DNET = dysembryoplastic neuroepithelial tumors; ODG = oligodendroglioma.

| Patient | Age (y), sex | Pathology | Type of electrode | HFO rate (HFO/min) | Seizure outcome |
| --- | --- | --- | --- | --- | --- |
| sub-01 | 15, f | DNET (WHO I) | 4x8 hd-ECoG | 6.0 | Good |
| sub-02 | 7, f | FCD2b | 4x8 hd-ECoG | 3.5 | Good |
| sub-03 | 17, m | Sturge Weber syndrome | 4x8 hd-ECoG | 2.0 | Good |
| sub-04 | 22, m | Ganglioglioma (WHOI) | 4x8 hd-ECoG | 709 | Good |
| sub-05 | 20, f | FCD2a | 4x8 hd-ECoG | 13.2 | Good |
| sub-06 | 3, f | Sturge Weber syndrome | 4x8 hd-ECoG | 31.7 | Poor |
| sub-07 | 34, m | Astrocytoma (WHOII) | 2x14 hd-ECoG | 1.3 | Good |
| sub-08 | 23, f | FCD2a | 4x8 hd-ECoG | 22.4 | Good |
| sub-09 | 1, m | FCD2a | 1x4 standard ECoG | 1.8 | Good |
| sub-10 | 15, f | Sturge Weber syndrome | 1x6 standard ECoG | < 1 | Poor |
| sub-11 | 36, f | Ependymoma (WHO II) | 1x6 standard ECoG | < 1 | Good |
| sub-12 | 38, m | anaplastic astrocytoma (WHO III) | 1x6 standard ECoG | < 1 | Good |
| sub-13 | 42, f | cavernoma | 1x8 standard ECoG | 1.2 | Poor |
| sub-14 | 7, f | FCD2b | 1x6 standard ECoG | 1.8 | Good |
| sub-15 | 17, m | FCD3 | 1x6 standard ECoG | n.a. | Poor |
| sub-16 | 67, f | ODG(WHOII) | 1x4 standard ECoG | < 1 | Good |
| sub-17 | 3, f | FCD 2a | 1x6 standard ECoG | n.a. | Poor |
| sub-18 | 4, m | FCD 1a | 2x8 standard ECoG | 1.2 | Poor |
| sub-19 | 17, m | Hippocampal sclerosis, gliosis | 2x8 standard ECoG | < 1 | Poor |
| sub-20 | 4, m | FCD1c | 1x4 standard ECoG | 7.0 | Good |
| sub-21 | 41, m | cavernoma | 2x8 standard ECoG | < 1 | Poor |
| sub-22 | 5, f | Fibrillary astrocytoma (WHOII) | 1x4 standard ECoG | < 1 | Good |

Supplementary Table 2. Seizure outcome of the patients in the validation set and their outcome classification. The outcome prediction yields the elements of the confusion matrix for the validation set (Table 1).^16^ TP = true positive, TN = true negative, FP = false positive, FN = false negative, Good = outcome Engel 1A, Poor = outcome Engel 1B–4, f = Female, m = male, MTS = mesial temporal sclerosis, MCD = mild cortical dysplasia, FCD = focal cortical dysplasia, DNET = dysembryoplastic neuroepithelial tumor, TSC = Tuberous sclerosis.

| Patient | Age (y), sex | Pathology | #Total Bipolar channels | Neuromorphic #HFO (#channels) | Seizure outcome | Outcome prediction |
| --- | --- | --- | --- | --- | --- | --- |
| sub-01 | 12, f | FCD 2b | 16 | - | Good | TN |
| sub-03 | 35, f | MTS (Wyler gr 4) | 15 | - | Good | TN |
| sub-05 | 44, m | TSC | 11 | 28 (10 ch) | Poor | TP |
| sub-06 | 41, m | FCD 2b | 9 | 5 (4 ch) | Poor | TP |
| sub-07 | 37, f | Cavernoma | 15 | 80 (11 ch) | Poor | TP |
| sub-09 | 12, f | Gliosis | 16 | 2 (2 ch) | Good | TN |
| sub-14 | 24, f | DNET (WHO gr 1) | 15 | - | Good | TN |
| sub-18 | 11, m | MCD | 14 | - | Good | TN |
| sub-20 | 25, m | developmental venous anomalies+ reactive changes | 17 | 9 (9 ch) | Good | TN |
| sub-21 | 34, m | Cavernoma | 15 | 1 (1 ch) | Good | TN |
| sub-23 | 3, f | DNET (WHO gr 1) | 12 | 2 (2 ch) | Poor | FN |
| sub-24 | 46, f | Iatrogenic cortical lesion - Hippocampal damage | 13 | 21 (10 ch) | Good | FP |
| sub-25 | 16, m | Ganglioglioma  (WHO gr 2) | 23 | - | Good | TN |
| sub-26 | 28, f | Ganglioglioma  (WHO gr 2) | 19 | 10 (8 ch) | Poor | TP |
| sub-27 | 20, m | Ganglioglioma  (WHO gr 2) | 16 | 1 (1 ch) | Poor | FN |
| sub-29 | 35, m | Suspected low-grade glioma | 15 | - | Good | TN |
| sub-30 | 46, f | Cavernoma +gliosis | 14 | 1 (1 ch) | Poor | FN |
| sub-31 | 15, m | Ganglioglioma (WHO gr 1-2) | 15 | - | Poor | FN |
| sub-33 | 31, m | MTS (Wyler gr 4) | 20 | - | Poor | FN |
| sub-36 | 40, f | MTS (Wyler gr 3) | 36 | - | Good | TN |
| sub-37 | 13, f | FCD 2b | 24 | 1 (1 ch) | Poor | FN |
| sub-40 | 53, f | Adenocarcinoma | 13 | 13 (13 ch) | Poor | FN |
| sub-46 | 14, m | FCD 2b | 17 | - | Good | TN |
| sub-47 | 12, m | DNET (WHO gr 1) | 14 | - | Poor | FN |
| sub-49 | 2, m | Unspecific migratory disorder +reactive changes | 13 | 2 (2 ch) | Good | TN |
| sub-51 | 31, f | Ganglioglioma  (WHO gr 1) | 11 | - | Good | TN |
| sub-52 | 8, m | FCD 2b | 14 | - | Good | TN |
| sub-56 | 14, m | DNET (WHO gr 1) | 25 | - | Good | TN |
| sub-60 | 11, f | Ganglioglioma  (WHO gr 1) | 16 | 2 (2 ch) | Good | TN |
| sub-61 | 49, f | MTS (Wyler gr 4) | 10 | - | Poor | FN |
| sub-62 | 15, f | MTS (Wyler gr 4) | 15 | 1 (1 ch) | Good | TN |
| sub-67 | 5, f | TSC | 14 | - | Good | TN |
| sub-68 | 29, m | Cavernoma | 8 | 1 (1 ch) | Poor | FN |
| sub-71 | 13, m | MTS (Wyler gr 4) | 16 | 200 (11 ch) | Poor | TP |
| sub-72 | 23, f | MTS (Wyler gr 4) | 18 | - | Poor | FN |
| sub-74 | 40, f | Cavernoma, MTS  (Wyler gr 4) | 16 | - | Poor | FN |
| sub-75 | 61, m | Unspecific migratory disorder | 16 | - | Good | TN |
| sub-76 | 13, m | FCD 2b /TSC | 17 | 9 (2 ch) | Poor | TP |
| sub-78 | 14, f | DNET (WHO gr 1) | 25 | - | Good | TN |
| sub-81 | 16, f | Ganglioglioma  (WHO gr 1) | 13 | - | Poor | FN |
| sub-83 | 1, f | Diffuse glioma  (WHO gr 2) | 14 | 4 (4 ch) | Poor | FN |
| sub-85 | 20, f | Pleiomorphic xantho-astrocytoma (WHO gr 2) | 19 | - | Good | TN |
| sub-86 | 6, f | Gliosis | 23 | - | Good | TN |
| sub-87 | 13, f | DNET | 14 | 1 (1 ch) | Poor | FN |
| sub-88 | 17, m | FCD 2b | 23 | - | Good | TN |
| sub-89 | 6, m | Astrocytoma (WHO gr 2) | 21 | - | Good | TN |
| sub-90 | 3, f | FCD 1a | 17 | - | Good | TN |
| sub-91 | 12, m | DNET | 18 | - | Good | TN |
| sub-92 | 24, m | Ganglioglioma  (WHO gr 1) | 12 | 6 (3 ch) | Poor | TP |
| sub-93 | 28, m | Ganglioglioma (WHO gr 1) | 7 | - | Good | TN |
| sub-94 | 13, m | FCD 2b | 27 | - | Good | TN |
| sub-95 | 9, m | FCD 2b /TSC | 14 | 13 (2 ch) | Poor | TP |
| sub-99 | 16, m | MCD + gliosis | 7 | 2 (1 ch) | Poor | TP |
| sub-104 | 12, m | DNET (WHO gr 1) | 14 | - | Good | TN |
